# On the Convergence Rate for the Longest at Most *T*-Contaminated Runs of Heads

**DOI:** 10.3390/e27010033

**Published:** 2025-01-03

**Authors:** István Fazekas, Borbála Fazekas, László Fórián

**Affiliations:** 1 Faculty of Informatics, University of Debrecen, Kassai Street 26, 4028 Debrecen, Hungary; forian.laszlo@inf.unideb.hu; 2 Institute of Mathematics, University of Debrecen, Egyetem Square 1, 4032 Debrecen, Hungary; borbala.fazekas@science.unideb.hu

**Keywords:** coin tossing, longest head run, asymptotic distribution, rate of convergence, 60F05

## Abstract

In this paper, we study the usual coin tossing experiment. We call a run at most *T*-contaminated, if it contains at most *T* tails. We approximate the distribution of the length of the longest at most *T*-contaminated runs. We offer a more precise approximation than the previous one.

## 1. Introduction

Consider the usual coin tossing experiment. Let *p* be the probability of heads and q=1−p be the probability of tails. Here, *p* is a fixed number with 0<p<1. We toss a coin *N* times independently. We write 1 for heads and 0 for tails. Therefore, we consider independent identically distributed random variables $X_{1},X_{2},\cdots ,X_{N}$ with distribution $P(X_{i}=1)=p$ and $P(X_{i}=0)=q=1-p$, i=1,2,⋯,N.

Let *T* be a fixed non-negative integer. We shall study the length of at most *T*-contaminated (in other words, at most *T*-interrupted) runs of heads. It means that there are at most *T* zeros in an *m*-length sequence of ones and zeros.

There are several well-known results on the length of the pure head runs. Fair coins were studied in the paper of Erdos and Rényi [1]. Almost sure limit results for the length of the longest runs containing at most *T* tails were obtained in [2]. Földes [3] presented asymptotic results for the distribution of the number of *T*-contaminated head runs, the first hitting time of a *T*-contaminated head run having a fixed length, and the length of the longest *T*-contaminated head run. Móri [4] proved an almost sure limit theorem for the longest *T*-contaminated head run.

Gordon, Schilling, and Waterman [5] applied extreme value theory to obtain the asymptotic behaviour of the expectation and the variance of the length of the longest *T*-contaminated head run. Then, accompanying distributions were obtained for the length of the longest *T*-contaminated head run. Ref. [6] proved results on the accuracy of the approximation to the distribution of the length of the longest head run in a Markov chain.

In this paper, we follow the lines of Arratia, Gordon, and Waterman [7], where Poisson approximation was used to find the asymptotic behaviour of the length of the longest at most *T*-contaminated head run. We shall use the basic results presented in [7], and give a new approximation for the distribution of the length of the longest at most *T*-contaminated head run. We show that for T>0 the rate of the approximation in our new result is $O\left(1/{\left(\log\left(n\right)\right)}^{2}\right)$, where log denotes the logarithm to base 1/p. Here and in what follows, f(n)=O(h(n)) means that f(n)/h(n) is bounded as n→∞. We shall see that for T>0 the rate of the approximation offered by [7] is $O\left(\log(\log(n))/\log(n)\right)$, so our result considerably improves the former result. In our opinion the much better rate $O\left(\log(n)/n\right)$ presented without detailed proof in [7] is just a misprint, that is true only for T=0. The main result is Theorem 1. For completeness, we give a proof of the former result, see Proposition 1. In Section 4, we present some simulation results supporting our theorem.

For T=1 and T=2, our result is the same as our former result in [8], where a powerful lemma by Csáki, Földes and Komlós [9] was used in the proof.

## 2. The Approximation of Arratia, Gordon, and Waterman

Using the notation of [7], let $S_{i}=X_{1}+\cdots +X_{i}$, and let $S_{n,t}$ be the largest increment in the sequence $S_{i}$ in *t* steps; more precisely, $S_{n,t}$ is the maximal number of heads in a window of length *t* starting in the first *n* tosses. Let $R_{n}\left(T\right)$ be the length of the longest at most *T*-interrupted runs of heads starting in the first *n* tosses. (One can see that $R_{n}\left(T\right)$ is the length of the longest precisely *T*-interrupted runs of heads starting in the first *n* tosses.) Then,

$$\left\{R_{n}\left(T\right)<t\right\}=\left\{S_{n,t}<t-T\right\}.$$

According to Theorem 1 of [7], for the distribution of $S_{n,t}$, we have the following approximation. For positive integers *n*, *s*, and *t* with s≤t and s/t>p,

(1) $$|P\left(S_{n,t}<s\right)-e^{-EW}|\leq 7tP\left(X_{1}+\cdots +X_{t}=s\right)+P\left(X_{1}+\cdots +X_{t}>s\right),$$

(2) $$\begin{array}{c} e^{-n\left(\frac{s}{t}-p\right)P\left(X_{1}+\cdots +X_{t}=s\right)}\cdot e^{-2n\left(1-\frac{s}{t}\right)P\left(X_{1}+\cdots +X_{t}=s\right)P\left(X_{1}+\cdots +X_{t}>s\right)}\\ \leq e^{-EW}\leq \;e^{-n\left(\frac{s}{t}-p\right)P\left(X_{1}+\cdots +X_{t}=s\right)}. \end{array}$$

In the above inequalities EW is the expectation of the random variable *W* defined in [7]. We shall use inequalities (1) and (2) with s=t−T. Using notation $\alpha =n\left(\frac{s}{t}-p\right)P\left(X_{1}+\cdots +X_{t}=s\right)$ and $\beta =2n\left(1-\frac{s}{t}\right)P\left(X_{1}+\cdots +X_{t}=s\right)P\left(X_{1}+\cdots +X_{t}>s\right)$, the above inequality is of the form

(3) $$e^{-\alpha }e^{-\beta }\leq e^{-EW}\leq e^{-\alpha }.$$

In this paper, the approximation of $e^{-\alpha }$ will serve as the main term.

Now, we shall analyse that approximation of $R_{n}\left(T\right)$ which was proposed in [7]. The centering constant in [7] is

(4) $$c_{n}\left(T\right)=\log n+T\log\log n-\log\left(T!\right)+\log\left(q^{T+1}p^{-T}\right).$$

Let *x* be a fixed number so that $c_{n}\left(T\right)+x=t$ is an integer. We want to estimate $P\left(R_{n}\left(T\right)-c_{n}\left(T\right)<x\right)=P\left(S_{n,t}<t-T\right)$. In the following we shall use both exp(x) and $e^{x}$ for the usual exponential function.

**Proposition** **1.** *Let $\left[c_{n}\left(T\right)\right]$ be the integer part of $c_{n}\left(T\right)$ and $\left\{c_{n}\left(T\right)\right\}=c_{n}\left(T\right)-\left[c_{n}\left(T\right)\right]$ be its fractional part.*

*If T=0, then for any integer l,*

(5) $$P\left(R_{n}\left(T\right)-\left[c_{n}\left(0\right)\right]<l\right)=\exp\left(-p^{l-\{c_{n}\left(0\right)\}}\right)\left(1+O\left(\frac{\log n}{n}\right)\right).$$

*If T>0, then for any integer l,*

(6) $$P\left(R_{n}\left(T\right)-\left[c_{n}\left(T\right)\right]<l\right)=\exp\left(-p^{l-\{c_{n}\left(T\right)\}}\right)\left(1+O\left(\frac{\log\log n}{\log n}\right)\right).$$

**Remark** **1.** *In Corollary 3 of [7], the same remainder term $O\left(\frac{\log n}{n}\right)$ is given for the case T>0, too. However, in our opinion, it contains only a part of the remainder terms.*

**Proof** **Proposition** **1.** As our remainder term and the remainder term offered by [7] are different, we give the details of the more or less simple calculation. First, we calculate the right hand side of inequality (1) for s=t−T and $t=c_{n}\left(T\right)+x$, where *x* is chosen so that *t* is an integer.

P(X1+⋯+Xt=t−T)=tTpt(q/p)T≤κ(logn)T(1/p)logn+Tloglogn=O1n.

Here and in what follows, κ is an appropriate finite positive constant. Therefore,

$$7tP\left(X_{1}+\cdots +X_{t}=t-T\right)=O\left(\frac{\log n}{n}\right).$$

For T>0, we have

P(X1+⋯+Xt>t−T)≤Ttt−T+1pt−T+1≤κtT−1pt≤κ(logn)T−1n(logn)T=O1nlogn.

So we obtain

(7) $$|P\left(S_{n,t}<t-T\right)-e^{-EW}|=O\left(\frac{\log n}{n}\right).$$

This last formula is valid for T=0, too.

Now, we turn to the other parts of the approximation. First, consider T=0. Then, the main term of the approximation, i.e., $e^{-\alpha }$ in Formula (3) is

$$e^{-\alpha }=e^{-n\left(\frac{t}{t}-p\right)P\left(X_{1}+\cdots +X_{t}=t\right)}=e^{-p^{-\log(nq)+t}}.$$

We have to approximate $P(R_{n}\left(0\right)-\left[c_{n}\left(0\right)\right]<l)$, where *l* is an integer, $c_{n}\left(0\right)=\log n+\log q$, and [.] denotes the integer part. So, we should apply the previous equality with $t=[c_{n}\left(0\right)]+l$, so we obtain

$$e^{-\alpha }=e^{-p^{l-\{c_{n}\left(0\right)\}}},$$

where {.} denotes the fractional part. We see that, if T=0, then β=0, so in inequality (3), we have equality. So, for T=0, this part of the approximation is precise, i.e., the main term does not contain a remainder part.

Now, we consider the approximation of the main term for T>0.

e−α=e−nt−Tt−pP(X1+⋯+Xt=t−T)=e−nq−TttTqTpt−T.

Now, denote by *L* the base 1/p logarithm of the negative of the exponent, that is, L=logα. So,

L=logn+log(q−T/t)+log(t(t−1)⋯((t−T+1))−logT!+Tlogq+T−t.

We shall use $t=c_{n}\left(T\right)+x$. Applying Taylor’s expansion of the logarithm function, $\log\left(x_{0}+y\right)=\log x_{0}+\frac{y}{cx_{0}}-\frac{y^{2}}{2c{\tilde{x}}_{0}^{2}}$, where ${\tilde{x}}_{0}$ is between $x_{0}$ and $x_{0}+y$, and where c=ln(1/p), we obtain

L=logn+logq−Tcqt−O1t2+logtT−tT−1T2ctT+O1t2−logT!+Tlogq+T−t

=logn+Tlogt−1ctTq+T2+O1t2−logT!+(T+1)logq+T−t.

We insert $t=c_{n}\left(T\right)+x=\log n+T\log\log n+E$, where *E* is defined by the equation at hand so it does not depend on *n*. Using again Taylor’s expansions of the logarithm function as $\log\left(x_{0}+y\right)=\log x_{0}+\frac{y}{cx_{0}}-\frac{y^{2}}{2cx_{0}^{2}}+\frac{y^{3}}{3c{\tilde{x}}_{0}^{3}}$, where ${\tilde{x}}_{0}$ is between $x_{0}$ and $x_{0}+y$, and for the 1/t function, as $\frac{1}{x_{0}+y}=\frac{1}{x_{0}}-\frac{y}{x_{0}^{2}}+\frac{y^{2}}{{\tilde{x}}_{0}^{3}}$, where ${\tilde{x}}_{0}$ is between $x_{0}$ and $x_{0}+y$, we obtain

L=logn+Tloglogn+Tloglogn+Eclogn−(Tloglogn+E)22c(logn)2+O(loglogn)3(logn)3−1cTq+T21logn−Tloglogn+E(logn)2+O(loglogn)2(logn)3+O1t2−logT!+(T+1)logq+T−t.

Now, using $t=c_{n}\left(T\right)+x$ and inserting the value of $c_{n}\left(T\right)$, we obtain

$$L=-x+\frac{T^{2}\log\log n}{c\log n}+O\left(\frac{1}{\log n}\right),$$

which implies that

$$L=-x+O\left(\frac{\log\log n}{\log n}\right),$$

and this rate is not improvable. We remark that this relation is valid for T=1, too.

Therefore, by applying the Taylor series expansion $e^{y}=1+y+e^{\tilde{y}}\frac{y^{2}}{2}$ twice, where $\tilde{y}$ is between 0 and *y*, we obtain

(8) $$\begin{array}{c} e^{-\alpha }=e^{-{\left(1/p\right)}^{L}}=e^{-p^{x}}\left(1-\ln\left(\frac{1}{p}\right)\frac{T^{2}\log\log n}{c\log n}+O\left(\frac{1}{\log n}\right)\right) \end{array}$$

(9) $$\begin{array}{c} =e^{-p^{l-\{c_{n}\left(T\right)\}}}\left(1+O\left(\frac{\log\log n}{\log n}\right)\right), \end{array}$$

and this rate is not improvable.

Now, we consider the $e^{-\beta }$ part. Here,

β=2Tt∑i=t−T+1ttipiqt−intTpt−TqT

with $t=c_{n}\left(T\right)+x=\log n+T\log\log n+E$. The largest term in the above sum is the first one, and it is

tT−1ptqpT−1=O1nlogn.

Then,

tTpt−TqT=O1n.

Using Taylor’s expansion,

$$\frac{T}{t}=O\left(\frac{1}{\log n}\right).$$

So, $\beta =O(1/n{\left(\log n\right)}^{2})$, and

$$e^{-\beta }=1-O\left(\frac{1}{n{\left(\log n\right)}^{2}}\right).$$

Therefore,

(10) $$\begin{array}{c} e^{-\alpha }e^{-\beta }=e^{-p^{l-\{c_{n}\left(T\right)\}}}\left(1+O\left(\frac{\log\log n}{\log n}\right)\right)\left(1-O\left(\frac{1}{n{\left(\log n\right)}^{2}}\right)\right)\\ =e^{-p^{l-\{c_{n}\left(T\right)\}}}\left(1+O\left(\frac{\log\log n}{\log n}\right)\right). \end{array}$$

□

## 3. A New Approximation

**Theorem** **1.** *Let T≥1 be an integer. Let*

(11) $$\begin{array}{ccc} {\tilde{c}}_{n}\left(T\right) & = & \log(qn)+T\log(\log(qn))\\ & + & T^{2}\frac{\log(\log(qn))}{c\log(qn)}-\frac{T}{cq_{0}\log\left(qn\right)}-\frac{T^{3}}{2c}{\left(\frac{\log(\log(qn))}{\log(qn)}\right)}^{2}\\ & + & T^{2}\frac{\log(\log(qn))}{cq_{0}{\left(\log\left(qn\right)\right)}^{2}}+T^{3}\frac{\log(\log(qn))}{{\left(c\log\left(qn\right)\right)}^{2}}\\ & + & \left(T\log\left(\frac{q}{p}\right)-\log\left(T!\right)\right)\left(1+\frac{T}{c\log(qn)}-T^{2}\frac{\log(\log(qn))}{c{\left(\log\left(qn\right)\right)}^{2}}\right), \end{array}$$

*where log denotes the logarithm to base 1/p, c=ln(1/p), ln denotes the natural logarithm to base e, and $q_{0}=\frac{2q}{2+Tq-q}$. Let $\left[{\tilde{c}}_{n}\left(T\right)\right]$ denote the integer part of ${\tilde{c}}_{n}\left(T\right)$, while $\left\{{\tilde{c}}_{n}\left(T\right)\right\}$ denotes the fractional part of ${\tilde{c}}_{n}\left(T\right)$, i.e. $\left\{{\tilde{c}}_{n}\left(T\right)\right\}={\tilde{c}}_{n}\left(T\right)-\left[{\tilde{c}}_{n}\left(T\right)\right]$.*

*Then,*

(12) $$\begin{array}{ccc} & & P(R_{n}\left(T\right)-\left[{\tilde{c}}_{n}\left(T\right)\right]<l)\\ & & \;\;=\exp\left(-p^{\left(l-\left\{{\tilde{c}}_{n}\left(T\right)\right\}\right)\left(1-\frac{T}{c\log(qn)}+T^{2}\frac{\log(\log(qn))}{c{\left(\log\left(qn\right)\right)}^{2}}\right)}\right)\left(1+O\left(\frac{1}{{\left(\log n\right)}^{2}}\right)\right) \end{array}$$

*for any integer l, where f(n)=O(h(n)) means that f(n)/h(n) is bounded as n→∞.*

**Proof.**  We use the same approach as in the previous section. First, we calculate the right hand side of inequality (1) for s=t−T and $t={\tilde{c}}_{n}\left(T\right)+x$, where *x* is chosen so that *t* is an integer. As

$${\tilde{c}}_{n}\left(T\right)=\log\left(n\right)+T\log\left(\log\left(n\right)\right)+O\left(1\right),$$

we obtain

P(X1+⋯+Xt=t−T)=tTpt(q/p)T≤κ(logn)T(1/p)logn+Tloglogn=O1n.

Therefore,

$$7tP\left(X_{1}+\cdots +X_{t}=t-T\right)=O\left(\frac{\log n}{n}\right).$$

Similarly,

$$P\left(X_{1}+\cdots +X_{t}>t-T\right)\leq \kappa t^{T-1}p^{t}=O\left(\frac{1}{n\log n}\right).$$

So,

(13) $$|P\left(S_{n,t}<t-T\right)-e^{-EW}|=O\left(\frac{\log n}{n}\right).$$

Now, we turn to the approximation of the main term $e^{-\alpha }$. Denote by *L* again the base 1/p logarithm of the negative of the exponent, so

$$\begin{array}{ccc} L & = & \log\alpha \\ & = & \log n+\log(q-T/t)+\log(t(t-1)\cdots ((t-T+1))-\log T!+T\log q+T-t. \end{array}$$

We shall apply it for $t={\tilde{c}}_{n}\left(T\right)+x$. Therefore,

$$\begin{array}{ccc} L & & \\ & = & \log\left(q-\frac{T}{t}\right)+\log n+\log\left(t^{T}-\frac{T(T-1)}{2}t^{T-1}+O\left(t^{T-2}\right)\right)-t\\ & & \;+\log({\left(q/p\right)}^{T})-\log\left(T!\right)\\ & = & \log\left(q-\frac{T}{t}\right)+\log n+\log\left(t^{T}\right)-\frac{\frac{T(T-1)}{2}t^{T-1}}{ct^{T}}+O\left(\frac{1}{t^{2}}\right)-t\\ & & \;+\log({\left(q/p\right)}^{T})-\log\left(T!\right)\\ & = & \log q-\frac{T}{cqt}+\log n+T\log t-\frac{\frac{T(T-1)}{2}}{ct}-t\\ & & \;+\log\left({\left(q/p\right)}^{T}\right)-\log\left(T!\right)+O\left(\frac{1}{{\left(\log n\right)}^{2}}\right)\\ & = & \log\left(qn\right)-\frac{T}{cq_{0}t}+T\log t-t+\log\left({\left(q/p\right)}^{T}\right)-\log\left(T!\right)+O\left(\frac{1}{{\left(\log n\right)}^{2}}\right), \end{array}$$

where we applied Taylor’s expansion of the log function up to the second order and used the notation $q_{0}=\frac{2q}{2+Tq-q}$.

Introduce notation

(14) $$\begin{array}{ccc} D & = & -\frac{T^{3}}{2c}{\left(\frac{\log(\log(qn))}{\log(qn)}\right)}^{2}+T^{2}\frac{\log(\log(qn))}{cq_{0}{\left(\log\left(qn\right)\right)}^{2}}+T^{3}\frac{\log(\log(qn))}{{\left(c\log\left(qn\right)\right)}^{2}}\\ & + & \left(T\log\left(\frac{q}{p}\right)-\log\left(T!\right)\right)\left(\frac{T}{c\log(qn)}-T^{2}\frac{\log(\log(qn))}{c{\left(\log\left(qn\right)\right)}^{2}}\right), \end{array}$$

(15) $$B=T^{2}\frac{\log(\log(qn))}{c\log(qn)}-\frac{T}{cq_{0}\log\left(qn\right)}+D$$

and

A=Tlog(log(qn))+B.

Then, $t={\tilde{c}}_{n}\left(T\right)+x={\tilde{c}}_{n}\left(T\right)+l-\left\{{\tilde{c}}_{n}\left(T\right)\right\}$, where *l* is an integer, so

$$t=T\log\left(\frac{q}{p}\right)-\log\left(T!\right)+\log\left(qn\right)+A+l-\left\{{\tilde{c}}_{n}\left(T\right)\right\}.$$

Inserting this value of *t* into the term −t of *L*, we obtain

$$\begin{array}{ccc} L & = & -\frac{T}{cq_{0}t}+T\log t-A-l+\left\{{\tilde{c}}_{n}\left(T\right)\right\}+O\left(\frac{1}{{\left(\log n\right)}^{2}}\right). \end{array}$$

Then, use Taylor’s expansion for the function 1/t to obtain

$$\begin{array}{ccc} L & = & -\frac{T}{cq_{0}\log\left(qn\right)}+T^{2}\frac{\log(\log(qn))}{cq_{0}{\left(\log\left(qn\right)\right)}^{2}}\\ & + & T\log\left(\log\left(qn\right)+T\log\left(\log\left(qn\right)\right)+B+\log\left({\left(q/p\right)}^{T}\right)-\log\left(T!\right)+l-\left\{{\tilde{c}}_{n}\left(T\right)\right\}\right)\\ & - & A-l+\left\{{\tilde{c}}_{n}\left(T\right)\right\}+O\left(\frac{1}{{\left(\log n\right)}^{2}}\right). \end{array}$$

Now, by Taylor’s expansion for the log(x) function, we obtain

$$\begin{array}{ccc} L= & - & \frac{T}{cq_{0}\log\left(qn\right)}+T^{2}\frac{\log(\log(qn))}{cq_{0}{\left(\log\left(qn\right)\right)}^{2}}+T\log\left(\log\left(qn\right)\right)\\ & + & \frac{T\left(T\log\left(\log\left(qn\right)\right)+B+\log\left({\left(q/p\right)}^{T}\right)-\log\left(T!\right)+l-\left\{{\tilde{c}}_{n}\left(T\right)\right\}\right)}{c\log(qn)}\\ & - & \frac{1}{2}\frac{T{\left(T\log\left(\log\left(qn\right)\right)+B+\log\left({\left(q/p\right)}^{T}\right)-\log\left(T!\right)+l-\left\{{\tilde{c}}_{n}\left(T\right)\right\}\right)}^{2}}{c{\left(\log\left(qn\right)\right)}^{2}}\\ & - & A-l+\left\{{\tilde{c}}_{n}\left(T\right)\right\}+O\left(\frac{1}{{\left(\log n\right)}^{2}}\right). \end{array}$$

Now, we can omit *B* from the quadratic term. Then, we apply A=Tlog(log(qn))+B, so we obtain

$$\begin{array}{ccc} L= & - & \frac{T}{cq_{0}\log\left(qn\right)}+\frac{T^{2}\log\left(\log\left(qn\right)\right)}{cq_{0}{\left(\log\left(qn\right)\right)}^{2}}+\frac{T^{2}\log\left(\log\left(qn\right)\right)}{c\log(qn)}\\ & + & \frac{T(\log\left({\left(q/p\right)}^{T}\right)-\log\left(T!\right))}{c\log(qn)}+\frac{T^{3}\log\left(\log\left(qn\right)\right)}{{\left(c\log\left(qn\right)\right)}^{2}}-\frac{T^{2}}{q_{0}{\left(c\log\left(qn\right)\right)}^{2}}+\frac{TD}{c\log(qn)}\\ & + & \frac{T(l-\left\{{\tilde{c}}_{n}\left(T\right)\right)\})}{c\log(qn)}-\frac{1}{2}\frac{T^{3}{\left(\log\left(\log\left(qn\right)\right)\right)}^{2}}{c{\left(\log\left(qn\right)\right)}^{2}}\\ & - & \frac{1}{2}\frac{T{\left(\log\left({\left(q/p\right)}^{T}\right)-\log\left(T!\right)+l-\left\{{\tilde{c}}_{n}\left(T\right)\right\}\right)}^{2}}{c{\left(\log\left(qn\right)\right)}^{2}}\\ & - & \frac{2T}{2}\frac{T\log\left(\log\left(qn\right)\right)\left(\log\left({\left(q/p\right)}^{T}\right)-\log\left(T!\right)+l-\left\{{\tilde{c}}_{n}\left(T\right)\right\}\right)}{c{\left(\log\left(qn\right)\right)}^{2}}\\ & - & B-l+\left\{{\tilde{c}}_{n}\left(T\right)\right\}+O\left(\frac{1}{{\left(\log n\right)}^{2}}\right)\\ = & & \left(l-\left\{{\tilde{c}}_{n}\left(T\right)\right\}\right)\left(\frac{T}{c\log(qn)}-\frac{T^{2}\log\left(\log\left(qn\right)\right)}{c{\left(\log\left(qn\right)\right)}^{2}}-1\right)+O\left(\frac{1}{{\left(\log n\right)}^{2}}\right). \end{array}$$

So,

$$e^{-\alpha }=e^{-p^{\left(l-\left\{{\tilde{c}}_{n}\left(T\right)\right\}\right)\left(1-\frac{T}{c\log(qn)}+\frac{T^{2}\log\left(\log\left(qn\right)\right)}{c{\left(\log\left(qn\right)\right)}^{2}}\right)+O\left(\frac{1}{{\left(\log n\right)}^{2}}\right)}}.$$

Using Taylor’s expansion again,

$$e^{-\alpha }=e^{-p^{\left(l-\left\{{\tilde{c}}_{n}\left(T\right)\right\}\right)\left(1-\frac{T}{c\log(qn)}+\frac{T^{2}\log\left(\log\left(qn\right)\right)}{c{\left(\log\left(qn\right)\right)}^{2}}\right)}}\left(1+O\left(\frac{1}{{\left(\log n\right)}^{2}}\right)\right).$$

Now, turn to the $e^{-\beta }$ part, where

β=2Tt∑i=t−T+1ttipiqt−intTpt−TqT

and $t={\tilde{c}}_{n}\left(T\right)+x$. Simple calculations shows that $\beta \leq \kappa (1/n{\left(\log n\right)}^{2})$, and so

$$e^{-\beta }=1+O\left(\frac{1}{n{\left(\log n\right)}^{2}}\right).$$

Therefore,

$$e^{-\alpha }e^{-\beta }=e^{-p^{\left(l-\left\{{\tilde{c}}_{n}\left(T\right)\right\}\right)\left(1-\frac{T}{c\log(qn)}+\frac{T^{2}\log\left(\log\left(qn\right)\right)}{c{\left(\log\left(qn\right)\right)}^{2}}\right)}}\left(1+O\left(\frac{1}{{\left(\log n\right)}^{2}}\right)\right).$$

□

## 4. Simulation Results

We performed several computer simulation studies for certain fixed values of *p* and *T*. Here, we present the results of three simulations. The length of each simulated sequence was $N=10^{6}$, and s=2000 was the number of repetitions of the *N*-length sequences in each case. In each case, the number of contaminations was T=3.

**Figure 1:**
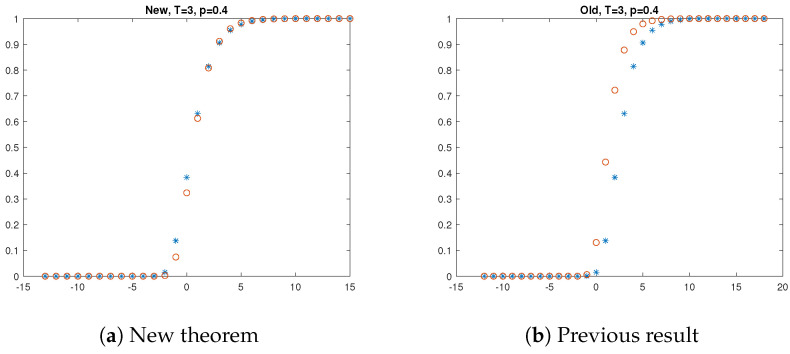
Longest at most T=3 contaminated run when p=0.4.

**Figure 2:**
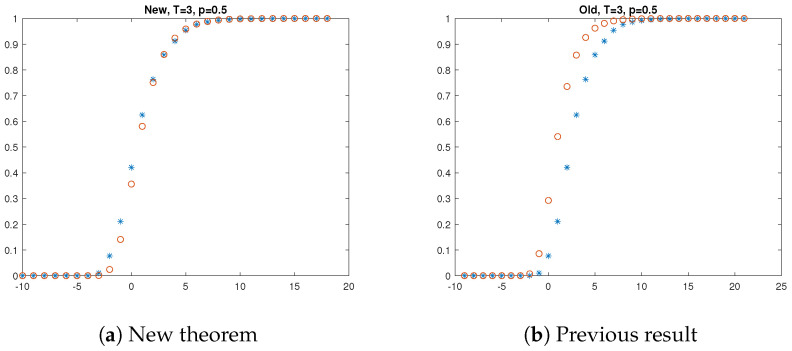
Longest at most T=3 contaminated run when p=0.5.

**Figure 3:**
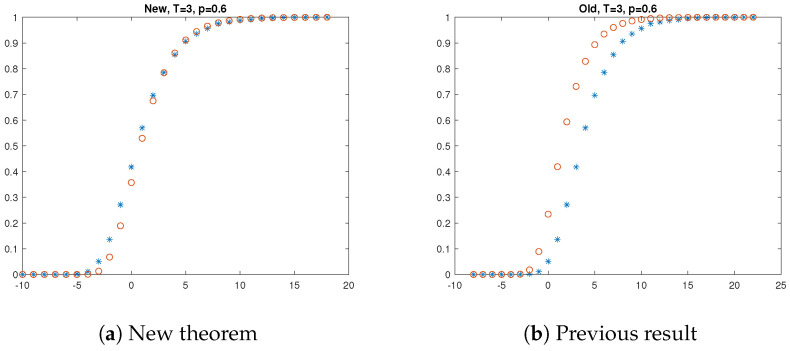
Longest at most T=3 contaminated run when p=0.6.

Figure 1, Figure 2 and Figure 3 present the results of the simulations. The left hand side of each figure shows the empirical distribution function of the longest at most *T*-contaminated run and its approximation suggested by our Theorem 1. The asterisk (i.e., ∗) denotes the result of the simulation, i.e., the empirical distribution of the longest at most *T*-contaminated run, and the circle (∘) denotes the approximation offered by Theorem 1. The right hand side of each figure shows the approximation by the former result. The asterisk denotes the result of the simulation again, and the circle (∘) denotes the approximation offered by Proposition 1. The simulation results support that our new theorem offers a better approximation than the previous one.

## 5. Discussion

We were able to obtain a practically applicable approximation for the distribution of the longest at most *T*-contaminated head-run. We presented both detailed mathematical proof and simulation evidence.

## Data Availability

The original contributions presented in this study are included in the article. Further inquiries can be directed to the corresponding author.
